# Altered resting-state connectivity in subjects at ultra-high risk for psychosis: an fMRI study

**DOI:** 10.1186/1744-9081-6-58

**Published:** 2010-10-11

**Authors:** Geumsook Shim, Jungsu S Oh, Wi Hoon Jung, Joon Hwan Jang, Chi-Hoon Choi, Euitae Kim, Hye-Yoon Park, Jung-Seok Choi, Myung Hun Jung, Jun Soo Kwon

**Affiliations:** 1 Department of Psychiatry, Seoul National University College of Medicine, 101 Daehak-no, Chongno-gu, Seoul, 110-744, Korea; 2 BK21 Division of Human Life Science, Seoul National University, Seoul, Korea; 3 Interdisciplinary Program in Brain Science, Seoul National University, Seoul, Korea; 4 Department of Radiology, National Medical Center, Seoul, Korea; 5 Department of Brain and Cognitive Sciences-World Class University Program, College of Natural Sciences, Seoul National University, Seoul, Korea

## Abstract

**Background:**

Individuals at ultra-high risk (UHR) for psychosis have self-disturbances and deficits in social cognition and functioning. Midline default network areas, including the medial prefrontal cortex and posterior cingulate cortex, are implicated in self-referential and social cognitive tasks. Thus, the neural substrates within the default mode network (DMN) have the potential to mediate self-referential and social cognitive information processing in UHR subjects.

**Methods:**

This study utilized functional magnetic resonance imaging (fMRI) to investigate resting-state DMN and task-related network (TRN) functional connectivity in 19 UHR subjects and 20 matched healthy controls. The bilateral posterior cingulate cortex was selected as a seed region, and the intrinsic organization for all subjects was reconstructed on the basis of fMRI time series correlation.

**Results:**

Default mode areas included the posterior/anterior cingulate cortices, the medial prefrontal cortex, the lateral parietal cortex, and the inferior temporal region. Task-related network areas included the dorsolateral prefrontal cortex, supplementary motor area, the inferior parietal lobule, and middle temporal cortex. Compared to healthy controls, UHR subjects exhibit hyperconnectivity within the default network regions and reduced anti-correlations (or negative correlations nearer to zero) between the posterior cingulate cortex and task-related areas.

**Conclusions:**

These findings suggest that abnormal resting-state network activity may be related with the clinical features of UHR subjects. Neurodevelopmental and anatomical alterations of cortical midline structure might underlie altered intrinsic networks in UHR subjects.

## Background

The 'default mode' is a term first coined by Raichle et al [1] to describe resting-state brain function and may be defined as a baseline condition of brain activity. The default mode network (DMN) refers to a set of functionally and anatomically organized neural regions that are active during a behavioral resting state and deactivated or suppressed during task performance [1,2]. The DMN most commonly includes the medial prefrontal cortex (mPFC) extending to ventral anterior cingulate cortex (ACC), the posterior cingulate cortex (PCC) extending to the precuneus (Pcu), and the lateral parietal cortex (LPC) [1]. Midline structures within the DMN have been implicated in self-referential cognitive and emotional tasks [3,4] as well as spontaneous thought processes known as mind wandering [5]. Self-referential processing mediated by the so-called cortical midline structures (CMS) is assumed to be the core of what is referred to as 'the self' [6,7]. In contrast, lateral neocortical networks such as the dorsolateral prefrontal cortex (DLPFC) are active during tasks that demand attention and working memory [8,9]. Thus, these networks may be referred to, respectively, as 'task-negative' and 'task-positive' networks, which are negatively correlated or anti-correlated [8].

The ability to understand another individual's mental state, called mentalizing or social cognition [10], is also an important aspect of resting-state brain function [11,12]. Human beings have a predisposition to engage in self-referential thought or social cognition, and the inclination to engage in such activity at rest (when not performing a task) may be mediated by the default system of the brain; the DMN [13]. Certain regions within the medial frontal lobe, including the ACC and the lateral parietal lobule, have been shown to be related to social cognition [14,15]. Functional imaging studies consistently identify increases in medial prefrontal cortical activity during social cognition tasks and have suggested that among such regions, the mPFC plays a predominant role in social cognition [16,17].

The social brain hypothesis [18,19] postulates that schizophrenia is a disorder of functional and structural connectivity within areas thought to regulate social cognition, such as the fronto-temporal and fronto-parietal cortical networks. Functional disintegration of these networks in patients with schizophrenia has been observed during performance of several types of cognitive tasks [20-22]. In addition, patients with schizophrenia consistently show evidence of abnormal resting-state functional connectivity [23-26], task-induced deactivation of CMS, and anti-correlation between DMN and task-related network (TRN) areas [25-27] when compared with healthy controls.

Over the last decade, the pre-onset, or prodromal, phase of schizophrenia has attracted considerable attention among researchers. Youths who are considered to be putatively prodromal have been identified using established criteria [28,29], and research on the characteristics of individuals at ultra-high risk (UHR) for psychosis has been conducted by several high-risk clinics. Neuroimaging studies have revealed that prior to the onset of psychosis, UHR youths already have brain abnormalities similar to those present in patients with schizophrenia [30-35]. These UHR youths also exhibit wide-ranging neuropsychological deficits comparable to those in patients with schizophrenia, although to a lesser degree [36,37]. These deficits include impaired social functioning and related problems with social skills [38-40], which are significant predictors of psychosis [41]. Recent studies from our group found that UHR individuals perform significantly worse during theory of mind (ToM) tasks, which measure the ability to conceptualize the mental state, beliefs, and intentions of other individuals [42]. Another fundamental feature of the prodromal phase of schizophrenia is self-disturbance [43,44], which is considered to be a psychopathological marker of psychotic vulnerability [45].

Therefore, the functionality of the DMN in UHR subjects is of primary interest, as it holds the potential to reveal any abnormalities in the activity of neural substrates regulating self-referential and social cognitive processing. It is possible that alterations in DMN function contribute to social cognitive deficits, such as diminished ToM capabilities, and to social dysfunction, such as social withdrawal and impairment of life-role functioning. However, whether UHR subjects exhibit normal connectivity in the default network remains unknown. Thus, this study investigated DMN function in two carefully matched groups of UHR subjects and healthy controls. Because UHR subjects show impairments in social cognition and self-referential processing regulated by the DMN as well as in neurocognitive abilities moderated by the TRN, it is hypothesized that DMN and/or TRN functional connectivity is altered in UHR subjects compared with healthy controls.

## Methods

### Participants

A total of 23 subjects at UHR for psychosis and 39 healthy volunteers underwent a resting-state functional magnetic resonance imaging (fMRI) scan. Four UHR subjects were excluded after MRI scanning; one withdrew consent, another was assessed as having transitioned to psychosis prior to scanning, the third was scanned in an open-eye state, and the fourth showed excessive head motion. The remaining 19 UHR subjects fulfilled the following diagnostic criteria for at least one of three UHR groups according to the Comprehensive Assessment of At-Risk Mental States (CAARMS) instrument [29]: (1) attenuated psychosis group (n = 17), (2) brief limited intermittent psychotic symptoms (n = 0), (3) vulnerability group (n = 5). Three subjects fulfilled the criteria for groups (1) and (3) concurrently. From among the 38 healthy volunteers (one was excluded as scanned in an open-eye state), 20 age-and gender-matched subjects were selected for between-group comparisons.

UHR subjects were recruited from the Seoul Youth Clinic (for detailed recruitment procedure and clinical assessments, see Chung et al [42] and Shin et al [35]). Seven subjects reported a family history of psychotic disorders; three had one first-degree relative with schizophrenia. Five UHR subjects were taking one or two psychotropic medications at the time of scanning, including anxiolytics (n = 4) and atypical antipsychotics (n = 3). The mean prodromal period for the 19 UHR subjects was 2.0 years (± 1.9), and three subjects have since converted to psychosis during follow-up monitoring with a mean of 83.7 days (± 53.9) after fMRI scanning. The healthy controls were recruited from an internet advertisement and screened using the Structured Clinical Interview for DSM-IV, non-patient edition [46]. All reported no personal or familial (i.e., first-to third-degree biological relatives) history of psychiatric disorders.

Participants were excluded if they had any lifetime diagnosis of substance abuse or dependence, neurological disease or brain injury, evidence of significant medical illness, or IQ less than 70. Several subjects had participated in previous studies from our group (one UHR subject and one control participated in Chung et al [42], two UHR subjects and five controls in Shin et al [35]). All participants provided written informed consent, including parental consent for those younger than 18 years of age. This study was approved by the Institutional Review Board at Seoul National University Hospital, and all procedures were performed in accordance with the current version of the Declaration of Helsinki.

### Image acquisition

Functional images were acquired using a 1.5 T MAGNETOM Avanto scanner (Siemens, Erlangen, Germany). Whole brain functional scans during a behavioral resting state were acquired in 25 contiguous axial slices approximately parallel to the anterior-posterior commissure plane with interleaved multi-slice echo-planar imaging according to the following parameters: TR = 2.34 s, TE = 52 ms, field of view = 22 cm, flip angle = 90°, voxel size = 3.44 × 3.44 × 5 mm, slice thickness = 5 mm, no inter-slice gap. For each participant, a total of 120 volumes during 4.68 min were acquired. fMRI scanning was carried out in darkness, and the participants were explicitly instructed to keep their eyes closed, relax, and move as little as possible. T1-weighted high-resolution structural images using a magnetization-prepared rapid acquisition gradient echo (MPRAGE) sequence were acquired in 176 contiguous axial slices for co-registration and normalization of the echo-planar images to the Montreal Neurologic Institute (MNI) template. Imaging parameters for the structural images were as follows: TR = 1.16 s, TE = 4.76 ms, field of view 23 cm, flip angle 15°, voxel size = 0.45 × 0.45 × 0.90 mm, slice thickness = 0.9 mm, no inter-slice gap.

An average gap of 8.7 days (± 10.0) occurred between clinical evaluation and fMRI scanning for all participants.

### fMRI preprocessing

Functional imaging analysis was performed using SPM5 software (Wellcome Dept. of Imaging Neuroscience, London, UK: http://www.fil.ion.ucl.ac.uk/spm) and in-house software running under the MATLAB environment (Mathworks, Inc.). For each subject, the first four images were discarded to eliminate the non-equilibrium effects of magnetization. The remaining functional images were corrected for differences in slice acquisition timing, which was followed by realignment to the middle image in the initial scan to correct for inter-scan movement and to remove signals correlated with head motion. Spatial normalization into the standard MNI template was performed and then smoothed using a Gaussian kernel of 6 mm full-width half-maximum to account for residual inter-subject differences.

### fMRI analysis

#### First-order analysis

The intrinsic organization (i.e., functional connectivity map) was separately reconstructed in the UHR subjects and healthy controls using the PCC as a seed region [8] and then analyzing the functional connectivity pattern during rest. As a major research interest with regard to resting-state fMRI is in slow-changing temporal activation, the fMRI data were temporally band-pass filtered (0.01-0.08Hz) [25,47] using finite impulse response filter to control for low-frequency drift and high-frequency noise. Generally, fMRI time series suffer from spurious correlations induced by partial volume effects of white matter, cerebrospinal fluid, and whole-brain functional activations. To account for this, multiple nuisance regressors [48] were generated from the segmentation results of T1 MPRAGE image using SPM5 software. A threshold value of 0.8 was applied for these segmentation maps, and "pure" white matter and cerebrospinal fluid masks that were less contaminated by other kinds of tissue were then extracted. For the generation of a whole-brain mask, a skull-stripping module called Brain Extraction tool in MRIcro software http://www.sph.sc.edu/comd/rorden/mricro.html was used. Movement nuisance regressors were also estimated using the realignment parameters provided by SPM5 software. Subsequently, the nuisance regressors were fitted using the least squares method for the fMRI time series in each voxel and were then regressed out using equation 1.

(1) $$\begin{array}{l} y=H\mu +\varepsilon ,\;where\;H\text{=}\left[y\_csf\;y\_wm\;y\_wb\;tx\;ty\;tz\;rx\;ry\;rz\right]\\ \hat{\mu }={\left(H^{T}H\right)}^{-\text{1}}H^{T}t\\ \hat{\varepsilon }=y-H\hat{\mu } \end{array}$$

Here, *y *is the observed time series of fMRI, *H *is the estimated nuisance regressors (*y_csf, y_wm*, and *y_wb *denote the average fMRI time series of CSF, skeletal white matter, and whole brain, respectively. *tx*, *ty*, *tz and rx*, *ry*, *rz *are translational and rotational movement regressors, respectively), *μ *is the mixture (coefficients) matrix of the nuisance regressors, and *ε *is the residual time series that was extracted as the true fMRI activation free from nuisance confounds. Assuming normality of the residual signal, a least square estimator $\hat{\mu }$ of *μ *was used in order to estimate $\hat{\varepsilon }$, the estimator of true fMRI activation *ε *that has no nuisance confounds.

The PCC, utilized as a seed region, was labeled using a Brodmann area (BA) atlas available in MRIcro software, which refers to the bilateral posterior cingulate as BA 23. The blood oxygen level-dependent (BOLD) time series of the voxels within this seed region were averaged to generate the reference time series. A correlation map was produced by computing the correlation coefficients between the reference time series and the time series from all other brain voxels. The resulting rho-maps were converted to *z*-values using a Fisher's rho-to-*z *transform to improve normality. Statistical maps of the intrinsic networks for UHR subjects and healthy controls were created by entering the single-subject intrinsic network component into a voxel-wise one-sample *t*-test using a criterion of *p *< 0.001 (uncorrected for multiple comparisons) for each group. All results were depicted on the surface, which was extracted by FreeSurfer software using a canonical brain provided by MRIcro software. An image-processing technique termed maximum intensity projection was used to visualize the most significant voxel at each location between two brain surfaces (i.e., the white and pial surfaces, which are the boundaries between the white and gray matter and between the gray matter and cerebrospinal fluid, respectively).

#### Second-order analysis

The intrinsic networks for each subject from the first-level analysis were entered into a second-level random effects analysis using two-sample *t*-tests. Between-group comparisons of two intrinsic networks were restricted to the regions belonging to the intrinsic networks of control group with a threshold at *p *< 0.001 (uncorrected) and a cluster size greater than 50 voxels.

## Results

### Demographic and clinical characteristics

Demographic and clinical characteristics for each group are provided in Table 1. All participants were right-handed, and the two groups were statistically similar in terms of parental socioeconomic status and IQ. However, compared to UHR subjects, healthy controls had a significantly higher number of educational years (U = 84.00, *p *= 0.002**) and scored significantly higher in the Global Assessment of Functioning scale (GAF; U = 12.50, *p *< 0.001**) and the Social Functioning Scale (SFS; *t *= -4.80, *p *< 0.001**).

**Table 1:** Demographic and clinical characteristics of subjects

Variables	UHR subjects (n = 19)		Healthy controls (n = 20)		Analysis	
			
	Mean	SD	Mean	SD	*T/U/X ^2^*	*p*
Male/Females	11/8		11/9		0.03^a^	0.86
Age (yrs)	20.8	4.1	21.7	2.1	-0.77^b^	0.45
Handedness (R/L)^e^	19/0		20/0			
Parental SES	3.0	1.2	3.2	1.2	165.00^c^	0.63
Educational years	12.2	2.0	13.9	1.3	84.00^c^	0.002**
IQ^f^	109.2	17.7	106.4	12.7	176.50^c^	0.70
GAF	52.3	11.6	89.8	2.0	12.50^c^	< 0.001**
SFS^g^	100.39	10.21	115.09	6.76	-4.80^d^	< 0.001**
PANSS	57.4	14.6				
BPRS	43.5	8.6				
SAPS	11.6	8.4				
SANS	28.4	16.5				
CAARMS^h^	46.5	18.8				

^a^Pearson's chi-square test; ^b^Welch's t test; ^c^Mann-Whitney U test; ^d^Student's t test.

^e^Assessed using Annett hand preference questionnaire [62].

^f^Estimated by Korean-Wechsler Adult Intelligence Scale-Revised (K-WAIS-R) [63].

^g^Average scores of the seven subscales, each of which was standardized and normalized with a mean of 100 and a SD of 15.

^h^Scored by adding the intensity rating scores.

Data were not available for some participants in parental SES (control n = 1) and SFS (control n = 5).

**p < 0.01

UHR: ultra-high risk; SES: Hollingshead socioeconomic status (highest = 1, lowest = 5); IQ: intelligence quotient; GAF: Global Assessment of Functioning [64]; SFS: Social Functioning Scale [65]; PANSS: Positive and Negative Syndrome Scale [66]; BPRS: Brief Psychiatric Rating Scale (modified 24-item version, rating items 1-7) [67]; SAPS: Scale for the Assessment of Positive Symptoms [68]; SANS: Scale for the Assessment of Negative Symptoms [69]; CAARMS: Comprehensive Assessment of At-Risk Mental States.

### Functional connectivity of the DMN and TRN

**Figure 1:**
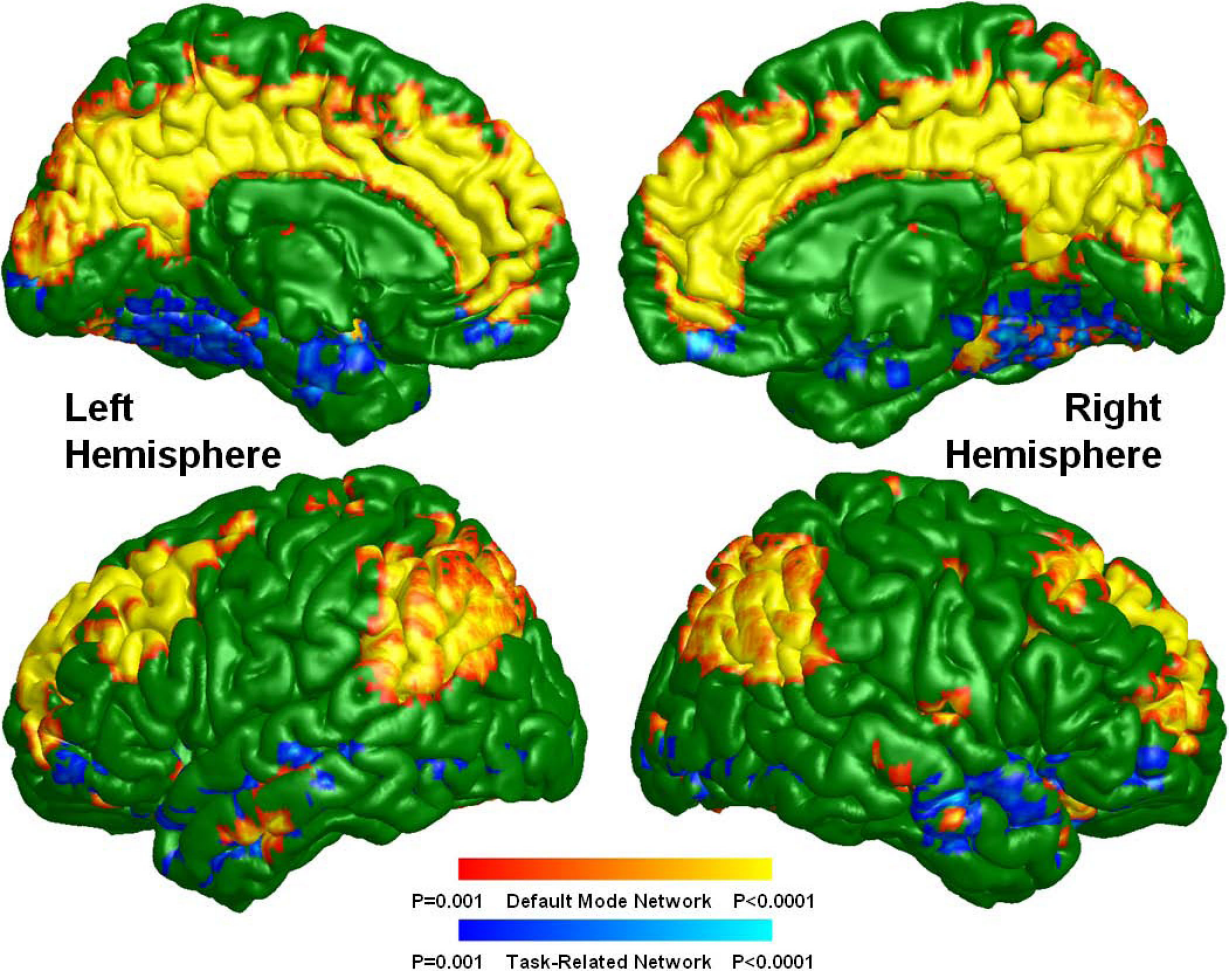
**Default mode and task-related maps for ultra-high risk subjects**. On a green background, the default mode network is highlighted in warm colors (red and yellow) and the task-related network is highlighted in cold colors (blue and light blue) depending on the p-value of one sample t-test.

**Figure 2:**
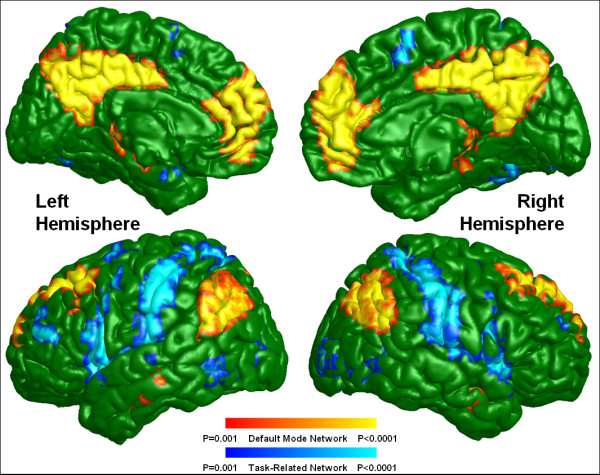
**Default mode and task-related maps for healthy controls**. The color codes for default mode and task-related networks are the same as for Figure 1.

**Figure 3:**
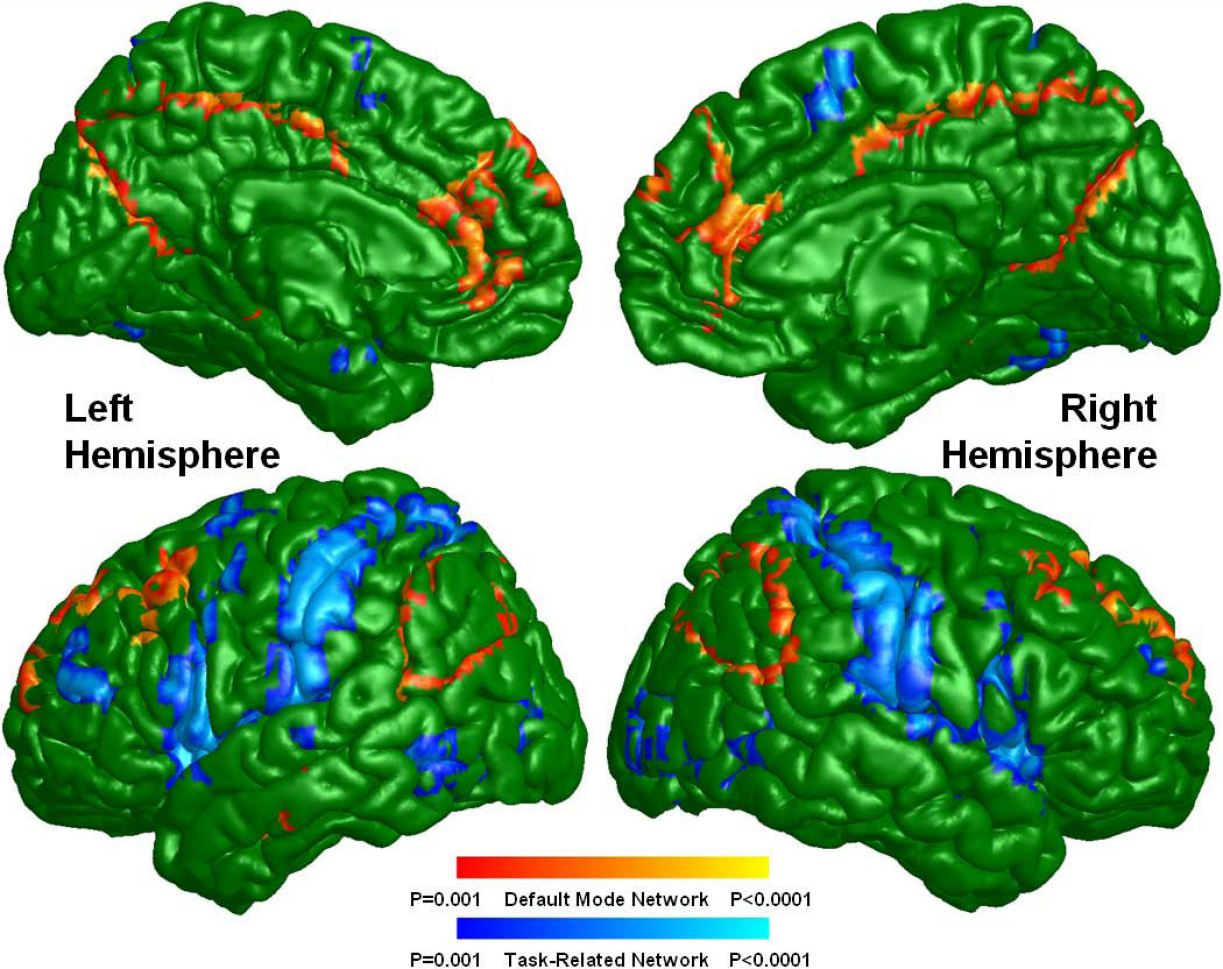
**Differences between UHR subjects and healthy controls in the resting state functional networks**. Default mode areas with increased connectivity in UHR subjects versus controls are shown in warm colors, and task-related areas with reduced anti-correlation in UHR subjects versus controls are shown in cold colors at the threshold of p < 0.001 (uncorrected) and cluster size greater than 50 voxels. UHR: ultra-high risk.

**Figure 4:**
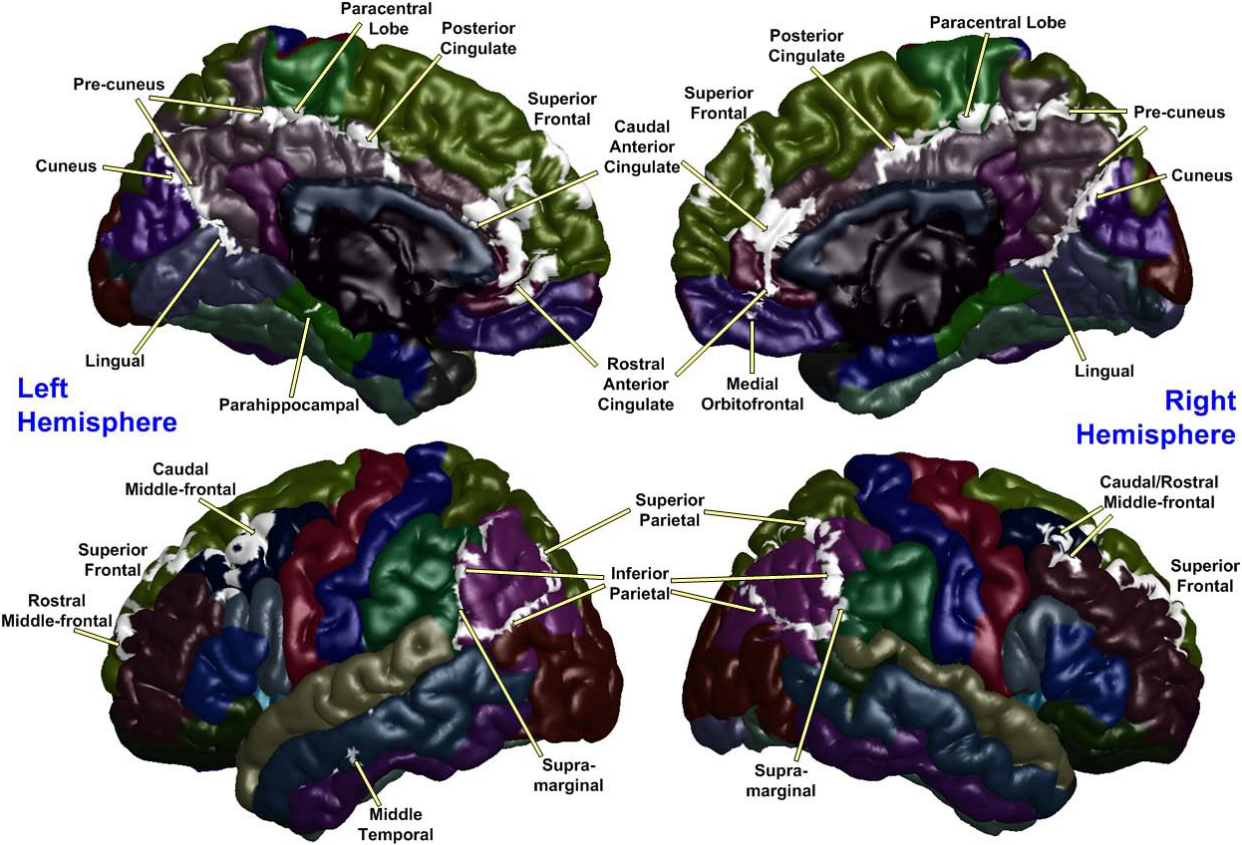
**Default mode areas in which ultra-high risk subjects showed significantly greater positive correlation with the posterior cingulate cortex than healthy controls**. Areas are thresholded at p < 0.001 (uncorrected) and cluster size greater than 50 voxels (highlighted in white).

Default mode activity was observed in brain regions previously defined as within the DMN (Figures 1 and 2) for both groups including the ACC, mPFC, Pcu, LPC, and the inferior temporal region. Although the default mode spatial maps look similar for UHR subjects and healthy controls, significant differences were observed in specific subregions of these areas (Figure 3). A two-sample *t*-test revealed that UHR subjects had significantly greater positive connectivity than did controls between the PCC seed region and other areas in the bilateral ACC, mPFC, Pcu, and LPC (cluster-level *p *< 0.001) (see Figure 4 for details). Healthy controls did not show greater positive connectivity than UHR subjects in any brain area.

**Figure 5:**
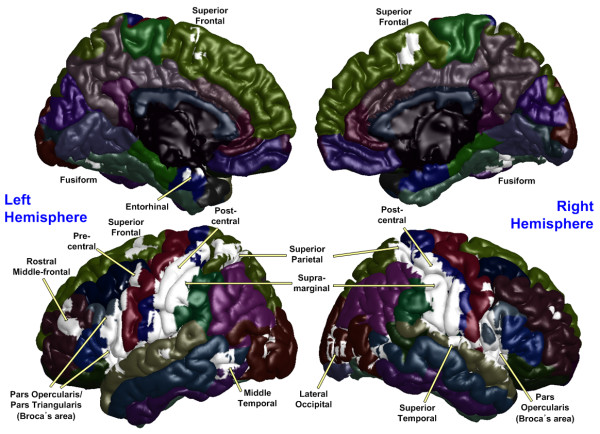
**Task-related areas in which ultra-high risk subjects showed significantly reduced anti-correlations with the posterior cingulate cortex compared to healthy controls**. Areas are thresholded at p < 0.001 (uncorrected) and cluster size greater than 50 voxels (highlighted in white).

Task-related or anti-correlated networks (Figures 1 and 2) and between-group differences (Figure 3) are also reported. The TRN areas are similar to previous reports [23,25] and include the DLPFC, supplementary motor area, the inferior parietal lobule, and middle temporal cortex. For the between-group comparison, the bilateral DLPFC, the inferior parietal lobule, middle temporal cortex, and left supplementary motor area are significantly more (i.e., farther from zero) anti-correlated with the PCC in controls than in UHR subjects (cluster-level *p *< 0.001) (see Figure 5 for details). Healthy controls did not exhibit a significantly reduced anti-correlation compared to UHR subjects.

## Discussion

To our knowledge, this is the first study directly investigating resting-state functional connectivity of UHR subjects versus healthy controls. During a resting state, UHR subjects exhibited hyperconnectivity within DMN regions as well as reduced anti-correlations between the PCC and TRN regions compared with healthy volunteers.

Despite inconsistent findings with respect to functional connectivity in resting-state networks of patients with schizophrenia, the current results agree with those of Whitfield-Gabrieli et al [26] and Zhou et al [25]. In these studies, the majority of participants were also in their early twenties and in the early phase of schizophrenia with acute psychotic symptoms. Chronic schizophrenia subjects with mild psychotic symptoms (mean duration ~10 years) exhibited reduced connectivity between areas of the default network [23]. Together, these results demonstrate that the DMN is hyperconnective during the prodromal and early psychotic stages of the disease, in which subjective discomfort and psychotic symptoms manifest and prevail, and that the DMN areas become progressively less synchronized, as aging and illness is progressing. A recent fMRI study utilizing the n-back task found that patients with chronic schizophrenia show reduced activation in the right DLPFC and other frontal areas, but greater activation in the ACC and mPFC compared with controls [49]. This finding demonstrates a failure to effectively deactivate the ACC and mPFC, and implies that those with schizophrenia are inefficient in their resource allocation when moving away from internal mentation to perform difficult tasks in the external world [49,50]. However, greater task-induced deactivation in the mPFC in schizophrenia subjects was also reported, and the magnitude of this change was associated with task performance [24,27]. Thus, the hyperconnectivity of the DMN and the reduced anti-correlation between the DMN and TRN may be involved in the impaired neurocognitive function of UHR subjects [36,37]. An inability to synchronize the modulation between two anti-correlated areas may also mediate these impairments.

In social phobia patients, a significantly lower deactivation in the posterior cingulate regions and Pcu was found during a face perception task compared with the resting condition [51]. This suggests that the failure of social phobia patients to deactivate the DMN plays an important role in their persistent fear of social situations and their self-focused attention. Social cognitive deficits, social anxiety (heighted sensitivity to interpersonal cues), and other social impairments are also common in UHR individuals. However, the present study focused solely on functional connectivity in midline default areas, and functional activation during ToM tasks was not measured. Future fMRI studies may be considered combining the resting-state and ToM tasks to investigate the relationship between altered midline default mode connectivity and impaired social cognition in UHR subjects.

The presence of structural abnormalities in the mPFC and ACC are well established in schizophrenia [52]. Similarly, UHR individuals also exhibit neurodevelopmental anomalies in midline brain structures such as the ACC and cavum septi pellucid [30,31]. In addition, compared to healthy controls, these subjects exhibit significant cortical thinning in the prefrontal cortex, ACC, and LPC [53] as well as reduced gray-matter volume of the PCC and Pcu [32]. These structural and functional abnormalities of the default mode-related areas may be significant in the altered functional connectivity of the default mode in UHR subjects in the current study.

Basic self-disturbances, or anomalies of self-experience, are a prominent feature during the prodromal stage of psychosis, and it is suspected this is the core disturbance in the emergence of schizophrenia-spectrum disorders [44,54,55]. In addition, reality distortion, or the impairment of socioemotional information processing, was associated with medial prefrontal cortical hyperactivity during viewing of aversive pictures in schizophrenia or schizoaffective patients [56]. From this perspective, hyperconnectivity of midline default areas in UHR subjects seems to be related to the so-called prodromal self-disturbance. However, further studies are necessary to apply a cognitive paradigm to subjective self-disturbance in UHR subjects to validate our assumption.

This study had several limitations. First, the interpretation of observed anti-correlations in resting state BOLD data is not straightforward [8]. Removal of spontaneous BOLD fluctuations common to the whole brain (the so-called global signal) mathematically mandates negative correlations, raising questions regarding the appropriateness of global signal regression and the interpretation of emerged anti-correlated networks [57]. However, anti-correlated networks were reported to be observed in the resting state without global regression [58], so cannot be fully explained as an artifact of global signal regression [57]. Although removal of the global signal facilitates the observation of true physiological relationships, great caution is required when comparing differences in anti-correlations between different clinical populations. Second, various conditions of internal mentation can influence resting-state brain activity, but the thoughts and feelings of subjects during the fMRI scanning period were not evaluated. Third, task paradigms including social cognition, self-reference, and subjective scales were not applied, limiting the analysis of the findings. Fourth, the reported conversion-to-psychosis rate (16%) is much lower than that of UHR cohorts (over 40%) tested in initial studies [59]. UHR status is not equivalent to being in the prodromal stage of schizophrenia or psychosis, so caution is needed in interpretation of these results. Finally, it is possible that medication could have influenced resting-state connectivity. However, 15 of 20 UHR subjects were drug-free, and the majority of previous studies investigating resting-state networks were conducted on medicated patients.

Several recent neuroimaging studies comparing UHR subjects who converted to psychosis (converters) with those who did not (non-converters) found that converters had less regional gray matter at baseline and greater gray matter reduction in longitudinal follow-ups than non-converters had [32,60,61]. Baseline ACC morphologic differences between converters and non-converters also predicted time-to-psychosis onset independent of symptomatology [33]. Similar to these comparison studies, longitudinal follow-up investigations of the current resting-state fMRI results may reveal differences within resting-state networks between converters and non-converters. This may provide valuable information about the properties of resting-state networks in terms of illness progression.

## Conclusions

The current findings demonstrate significant alterations (i.e., functional pathology) of resting-state networks in UHR subjects and suggest that hyperconnectivity of the DMN and reduced anti-correlation between the DMN and TRN may play an important role in the clinical features of these subjects. Regions previously identified to be abnormal in UHR subjects also showed clear abnormalities in the DMN. Further resting-state fMRI studies that include social cognition tasks and subjective rating scales are necessary to further validate the interpretation of the present results.

## Competing interests

The authors declare that they have no competing interests.

## Authors' contributions

GS was involved in conceiving the study, analyzing the data as well as writing the manuscript. JSO processed and analyzed the data, and helped to draft the manuscript. GS and JSO contributed equally to this work. WHJ participated in MRI acquisition, pre-processing and analysis, and assisted with interpretation of study findings. CHC aided in initial MRI data collection, supervised the MR scanning, and participated in the data analysis. JHJ, EK, HYP, JSC and MHJ recruited subjects, undertook clinical assessments of the participants and substantially contributed in writing the manuscript. JSK developed the Seoul Youth Clinic, designed and supervised the overall study, and reviewed the manuscript. All authors have read and approved the final manuscript.
